# The Assessment of C-shaped Canal Prevalence in Mandibular Second Molars Using Endodontic Microscopy and Cone Beam Computed Tomography: An In Vivo Investigation

**DOI:** 10.7759/cureus.62026

**Published:** 2024-06-09

**Authors:** Sabina Shaikh, Amey G Patil, Vedant U Kalgutkar, Sailee A Bhandarkar, Anuja Hakke Patil

**Affiliations:** 1 Department of Conservative Dentistry and Endodontics, DY Patil Dental School, Pune, IND; 2 Department of Restorative Dentistry, Rutgers School of Dental Medicine, Newark, USA; 3 Department of Diagnostic Sciences, Center for Temporomandibular Disorders and Orofacial Pain, Rutgers School of Dental Medicine, Newark, USA; 4 Department of Endodontics, Mahatma Gandhi Vidyamandir's Karmaveer Bhausaheb Hiray Dental College and Hospital, Maharashtra University of Health Sciences, Nashik, IND; 5 Department of Oral and Maxillofacial Radiology, Mahatma Gandhi Vidyamandir's Karmaveer Bhausaheb Hiray Dental College and Hospital, Maharashtra University of Health Sciences, Nashik, IND

**Keywords:** ethnic groups, root canal variations, root canal configuration, endodontic treatment, root canal anatomy, cone-beam computed tomography (cbct), mandibular second molar, c-shaped canal

## Abstract

Background

Understanding root canal anatomy variations, particularly C-shaped canals, is crucial for successful endodontic treatment. This study used clinical and radiographic methods to assess the prevalence and characteristics of C-shaped canals in mandibular second molars in Western Maharashtra.

Materials and methods

This prospective study was conducted in the western region of Maharashtra, India. The samples included patients requiring endodontic treatment for mandibular second molars. Clinical evaluation was conducted using a surgical endodontic microscope and cone beam computed tomography (CBCT) imaging. Inclusion and exclusion criteria ensured the selection of a focused and homogeneous sample. Data analysis included assessment of unilateral/bilateral occurrence, canal distribution, and cross-sectional characteristics.

Results

Out of 200 mandibular second molars, 7.5% exhibited C-shaped root canals, with no significant gender differences. Canal distribution varied across coronal, middle, and apical levels, with prevalent configurations being C1, C2, C3, and C4. No significant differences were observed in canal distribution based on root levels. No significant gender differences were found in the presence of grooves on the root surfaces.

Conclusion

This study provides valuable insights into the prevalence and characteristics of C-shaped canals in mandibular second molars in Western Maharashtra. Further research into histological and genetic aspects can enhance our understanding, leading to improved treatment strategies for complex root canal anatomy variations.

## Introduction

Understanding root canal anatomy and its variations among ethnic groups is crucial for successful endodontic treatment. The primary objective of endodontic therapy is to thoroughly clean and shape the root canal system and achieve complete four-dimensional obturation with an inert filling material [1]. A significant anatomical variation is the C-shaped root, resulting from the failure of Hertwig's epithelial root sheath to fuse on the lingual or buccal root surface, forming a C-shaped canal [2,3]. This variation is characterized by a fin or web connecting individual root canals, forming a 'C' shape at the root canal orifice [2,4]. C-shaped canals are most commonly found in mandibular second molars, followed by mandibular first premolars and molars, and maxillary first and second molars, with a higher prevalence in Asian populations [4]. Mandibular molars, frequently affected by decay, often require endodontic treatment, making them one of the most common teeth treated endodontically [5]. However, mandibular molars have a high extraction rate, mainly due to failed endodontic treatment [6]. Therefore, understanding root canal anatomy and variations is crucial for treatment success. This study aims to evaluate the proportion of C-shaped canals in mandibular second molars using an endodontic microscope and cone beam computed tomography (CBCT) in Western Maharashtra. The objectives include assessing the incidence of C-shaped canals in mandibular second molars and examining the cross-sectional characteristics of roots with C-shaped canals.

## Materials and methods

This prospective study was conducted at the Department of Conservative Dentistry and Endodontics, Krishna Institute of Medical Sciences Deemed University (KIMSDU), Malkapur, involving patients who visited the School of Dental Sciences for endodontic treatment of mandibular second molars over a period of 18 months. The Ethical Committee of Krishna Institute of Medical Sciences, Deemed University approved the study protocol, ensuring ethical standards in medical research (reference number KIMSDU/DR/573).

Inclusion criteria were patients requiring endodontic treatment for mandibular second molars with fully formed apices and teeth free from previous root canal treatments, posts, or crown restorations. Exclusion criteria included teeth with immature apices, root fractures, pre-existing external defects or cracks on the root surface, anatomical irregularities, caries involving the roots, root resorption, or teeth previously treated endodontically. These criteria ensured the selection of a focused and homogeneous sample for our investigation. The clinical evaluation involved the use of a surgical endodontic microscope and CBCT to precisely assess C-shaped canals. The methods employed aim to maintain the highest standards of clinical research and patient safety in accordance with ethical guidelines set by the Institutional Ethical Committee.

**Figure 1:**
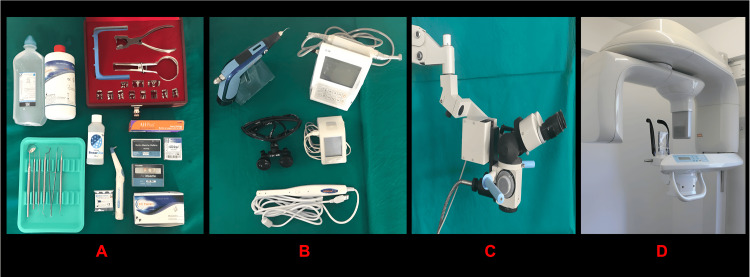
Endodontic treatment tools and equipment (A) Armamentarium used (mouth mirror, endodontic explorer, probe, Endo Access Kit, rubber dam kit, 2.5% sodium hypochlorite (NaOCl), 17% EDTA, K files (Mani Ink Japan); (B) Instruments used (Apex Locator (Root Zx mini, J Morita, USA), Endo motor (triauto mini, J Morita, USA), E and Q plus thermoplasticized obturation); (C) Surgical endodontic microscope; (D) CBCT Machine

The materials utilized included a mouth mirror, endodontic explorer, probe, Endo Access Kit, rubber dam kit, 2.5% sodium hypochlorite (NaOCl), 17% ethylenediaminetetraacetic acid (EDTA), K files (Mani Inc., Tochigi, Japan), Apex Locator (Root ZX mini, J Morita, Kyoto, USA), Endo motor (triauto mini, J Morita, Kyoto, USA), E and Q plus thermoplasticized obturation, surgical endodontic microscope, and CBCT machine (see Figure 1). An intraoral periapical radiographs (IOPA) were taken.

Procedure

**Figure 2:**
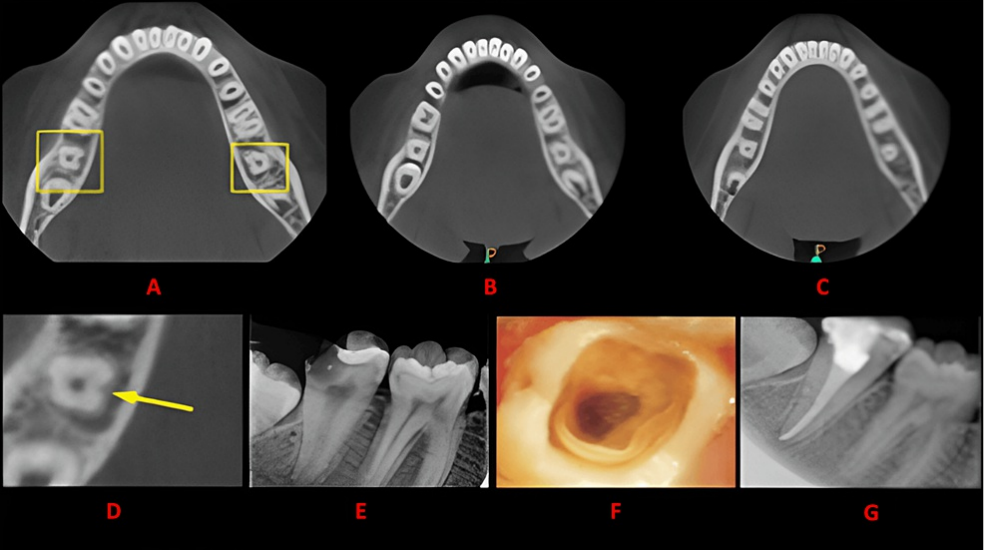
Case 1 The figure shows a cross-sectional analysis of the tooth under examination using CBCT. 'A' depicts the coronal third of the tooth, revealing a bilateral C-shaped canal presence; 'B' corresponds to the middle third, while 'C' represents the apical third; 'D' highlights the presence of a buccal groove; 'E' provides the postoperative radiograph; 'F' offers an intraoral image, and 'G' presents another view of the postoperative radiograph. CBCT: cone beam computed tomography

**Figure 3:**
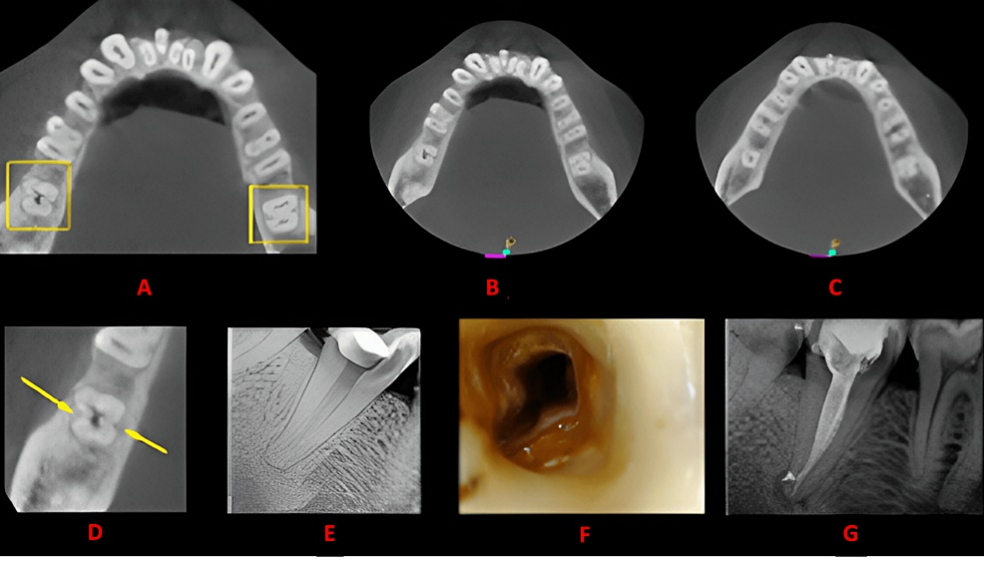
Case 2 This figure shows a cross-sectional analysis of the tooth under examination using CBCT. 'A' depicts the coronal third of the tooth, revealing a unilateral C-shaped canal presence; 'B' corresponds to the middle third, while 'C' represents the apical third; 'D' highlights the presence of a buccal and lingual groove; 'E' provides the postoperative radiograph; 'F' offers an intraoral image; and 'G' presents another view of the postoperative radiograph. CBCT: cone beam computed tomography

**Figure 4:**
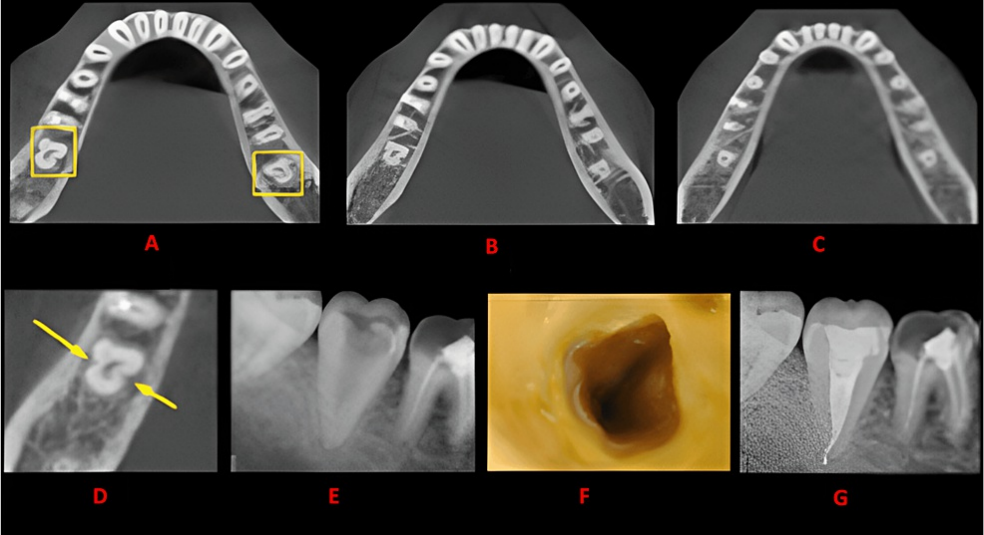
Case 3 This figure shows a cross-sectional analysis of the tooth under examination using CBCT. 'A' depicts the coronal third of the tooth, revealing a bilateral C-shaped canal presence; 'B' corresponds to the middle third, while 'C' represents the apical third; 'D' highlights the presence of a buccal and lingual groove; 'E' provides the postoperative radiograph; 'F' offers an intraoral image; and 'G' presents another view of the postoperative radiograph. CBCT: cone beam computed tomography

**Figure 5:**
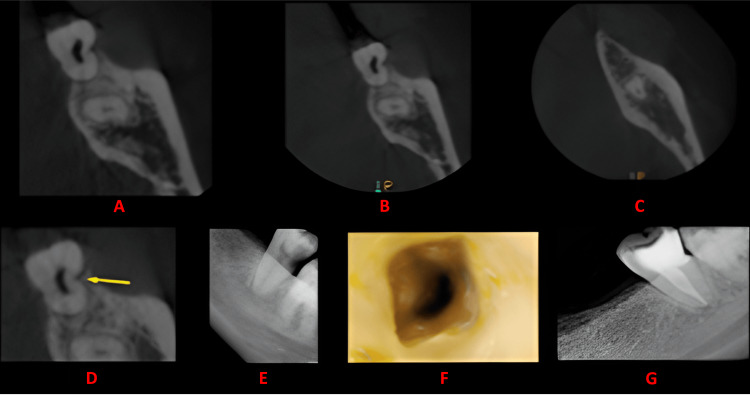
Case 4 This figure shows a cross-sectional analysis of the tooth under examination using CBCT. 'A' depicts the coronal third of the tooth, revealing a unilateral C-shaped canal presence; 'B' corresponds to the middle third, while 'C' represents the apical third; 'D' highlights the presence of a lingual groove; 'E' provides the postoperative radiograph; 'F' offers an intraoral image; and 'G' presents another view of the postoperative radiograph. CBCT: cone beam computed tomography

**Figure 6:**
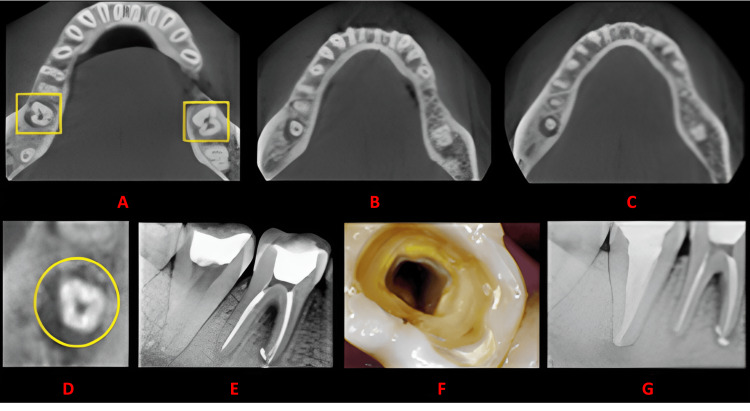
Case 5 This figure shows a cross-sectional analysis of the tooth under examination using CBCT. 'A' depicts the coronal third of the tooth, revealing a unilateral C-shaped canal presence; 'B' corresponds to the middle third, while 'C' represents the apical third; 'D' highlights the absence of any groove; 'E' provides the postoperative radiograph; 'F' offers an intraoral image; and 'G' presents another view of the postoperative radiograph. CBCT: cone beam computed tomography

**Figure 7:**
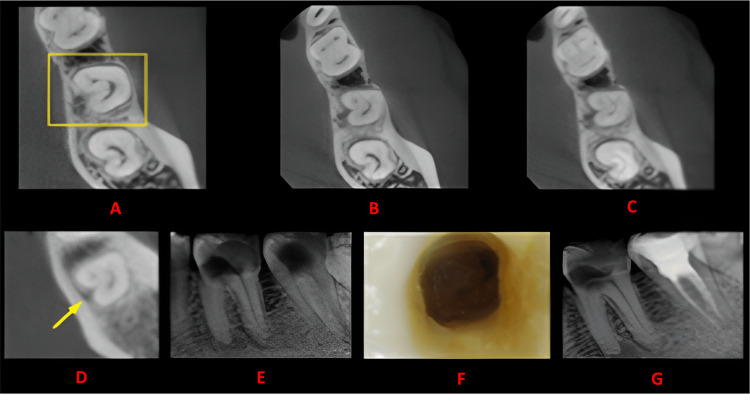
Case 6 This figure shows a cross-sectional analysis of the tooth under examination using CBCT. 'A' depicts the coronal third of the tooth, revealing a unilateral C-shaped canal presence; 'B' corresponds to the middle third, while 'C' represents the apical third; 'D' highlights the presence of a lingual groove; 'E' provides the postoperative radiograph; 'F' offers an intraoral image; and 'G' presents another view of the postoperative radiograph. CBCT: cone beam computed tomography

**Figure 8:**
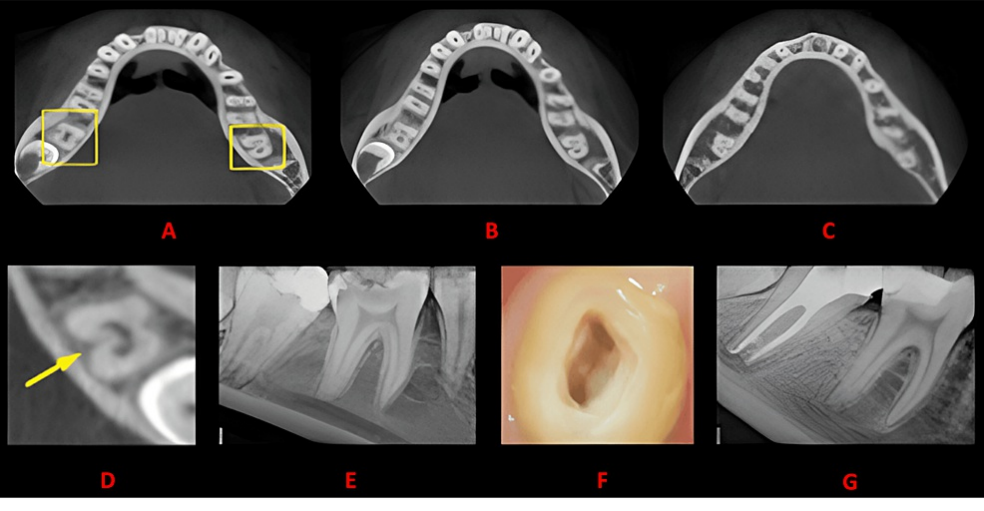
Case 7 This figure shows a cross-sectional analysis of the tooth under examination using CBCT. 'A' depicts the coronal third of the tooth, revealing a unilateral C-shaped canal presence; 'B' corresponds to the middle third, while 'C' represents the apical third; 'D' highlights the presence of a lingual groove; 'E' provides the postoperative radiograph; 'F' offers an intraoral image; and 'G' presents another view of the postoperative radiograph. CBCT: cone beam computed tomography

**Figure 9:**
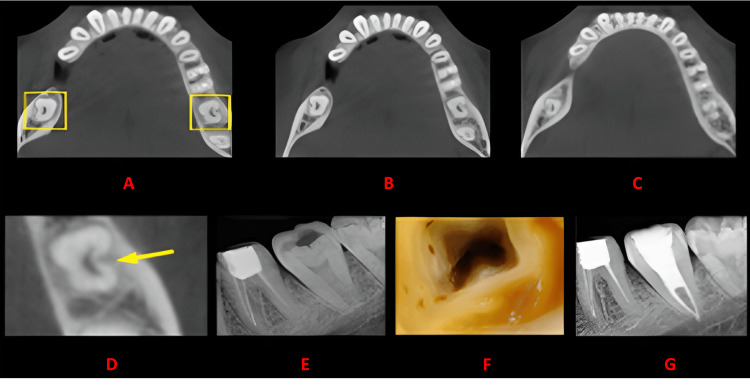
Case 8 This figure shows a cross-sectional analysis of the tooth under examination using CBCT. 'A' depicts the coronal third of the tooth, revealing a bilateral C-shaped canal presence; 'B' corresponds to the middle third, while 'C' represents the apical third; 'D' highlights the presence of a buccal groove; 'E' provides the postoperative radiograph; 'F' offers an intraoral image; and 'G' presents another view of the postoperative radiograph. CBCT: cone beam computed tomography

**Figure 10:**
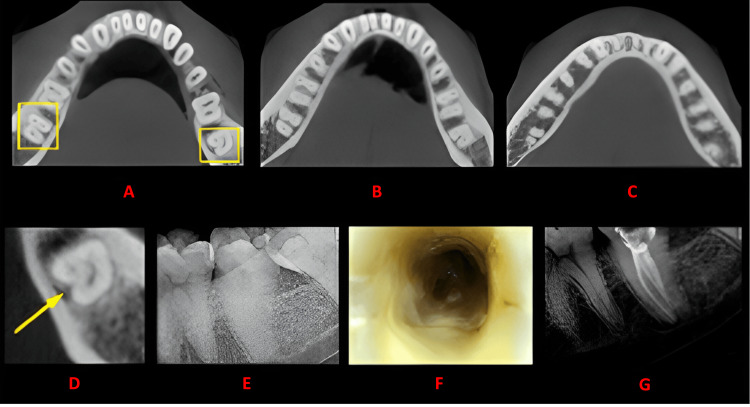
Case 9 This figure shows a cross-sectional analysis of the tooth under examination using CBCT. 'A' depicts the coronal third of the tooth, revealing a unilateral C-shaped canal presence; 'B' corresponds to the middle third, while 'C' represents the apical third; 'D' highlights the presence of a lingual groove; 'E' provides the postoperative radiograph; 'F' offers an intraoral image; and 'G' presents another view of the postoperative radiograph. CBCT: cone beam computed tomography

**Figure 11:**
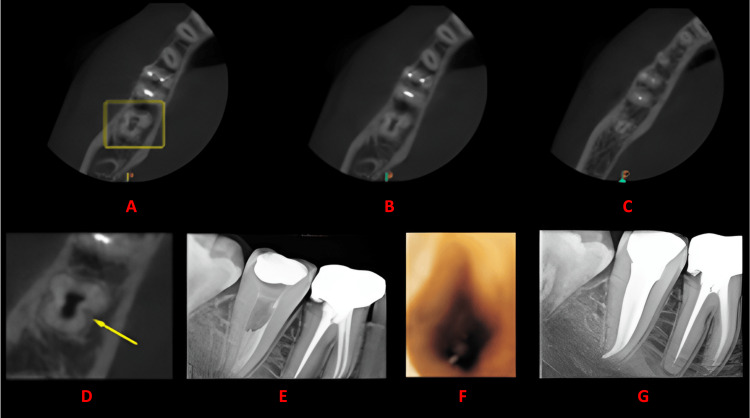
Case 10 This figure shows a cross-sectional analysis of the tooth under examination using CBCT. 'A' depicts the coronal third of the tooth, revealing a unilateral C-shaped canal presence; 'B' corresponds to the middle third, while 'C' represents the apical third; 'D' highlights the presence of a lingual groove; 'E' provides the postoperative radiograph; 'F' offers an intraoral image; and 'G' presents another view of the postoperative radiograph. CBCT: cone beam computed tomography

**Figure 12:**
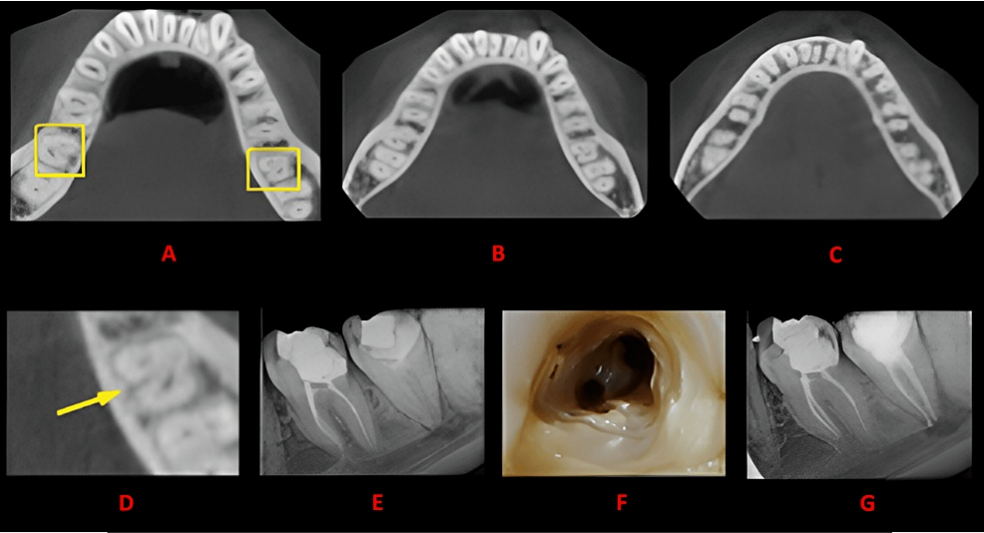
Case 11 This figure shows a cross-sectional analysis of the tooth under examination using CBCT. 'A' depicts the coronal third of the tooth, revealing a bilateral C-shaped canal presence; 'B' corresponds to the middle third, while 'C' represents the apical third; 'D' highlights the presence of a lingual groove; 'E' provides the postoperative radiograph; 'F' offers an intraoral image; and 'G' presents another view of the postoperative radiograph. CBCT: cone beam computed tomography

**Figure 13:**
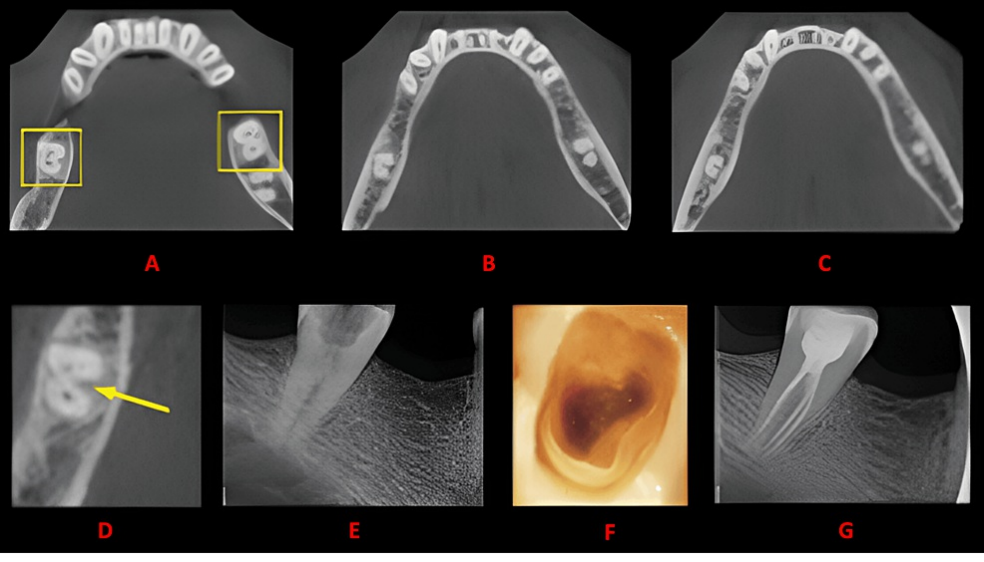
Case 12 This figure shows a cross-sectional analysis of the tooth under examination using CBCT. 'A' depicts the coronal third of the tooth, revealing a unilateral C-shaped canal presence; 'B' corresponds to the middle third, while 'C' represents the apical third; 'D' highlights the presence of a lingual groove; 'E' provides the postoperative radiograph; 'F' offers an intraoral image; and 'G' presents another view of the postoperative radiograph. CBCT: cone beam computed tomography

**Figure 14:**
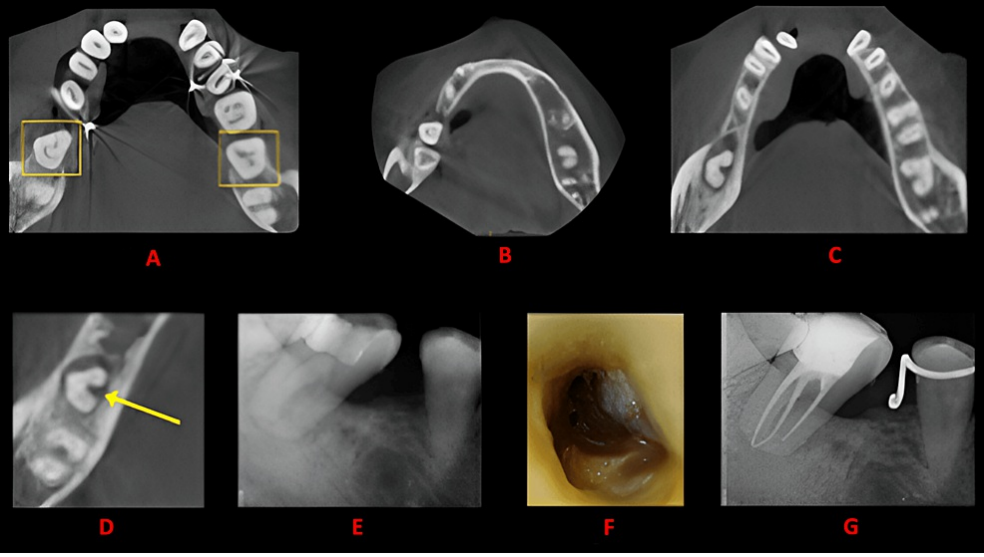
Case 13 This figure shows a cross-sectional analysis of the tooth under examination using CBCT. 'A' depicts the coronal third of the tooth, revealing a bilateral C-shaped canal presence; 'B' corresponds to the middle third, while 'C' represents the apical third; 'D' highlights the presence of a lingual groove; 'E' provides the postoperative radiograph; 'F' offers an intraoral image; and 'G' presents another view of the postoperative radiograph. CBCT: cone beam computed tomography

**Figure 15:**
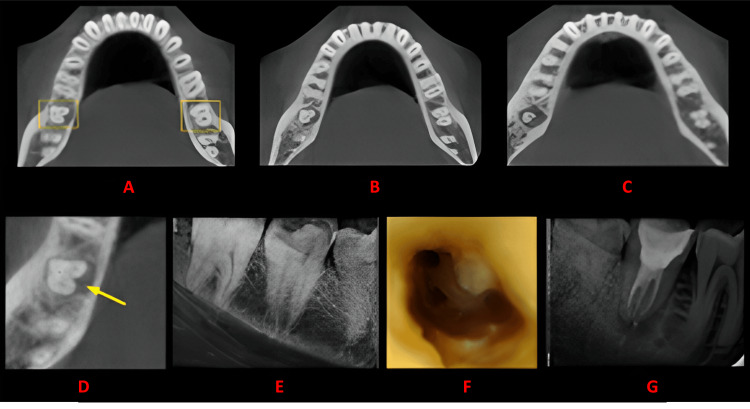
Case 14 This figure shows a cross-sectional analysis of the tooth under examination using CBCT. 'A' depicts the coronal third of the tooth, revealing a unilateral C-shaped canal presence; 'B' corresponds to the middle third, while 'C' represents the apical third; 'D' highlights the presence of a lingual groove; 'E' provides the postoperative radiograph; 'F' offers an intraoral image; and 'G' presents another view of the postoperative radiograph. CBCT: cone beam computed tomography

**Figure 16:**
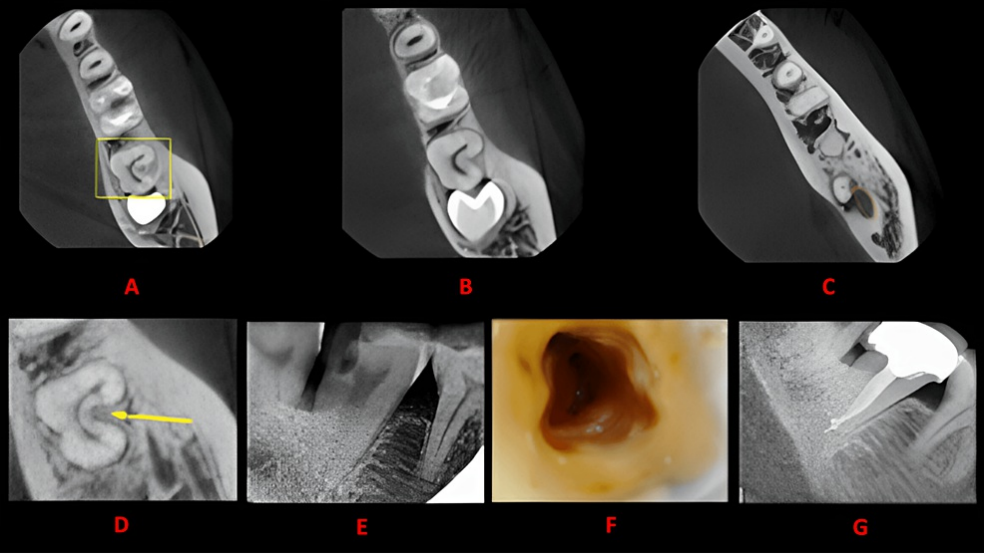
Case 15 This figure shows a cross-sectional analysis of the tooth under examination using CBCT. 'A' depicts the coronal third of the tooth, revealing a unilateral C-shaped canal presence; 'B' corresponds to the middle third, while 'C' represents the apical third; 'D' highlights the presence of a lingual groove; 'E' provides the postoperative radiograph; 'F' offers an intraoral image; and 'G' presents another view of the postoperative radiograph. CBCT: cone beam computed tomography

The evaluation of C-shaped canal systems in the permanent mandibular second molars of 100 patients, totaling 200 mandibular second molars, was performed clinically using Min's method, employing a surgical endodontic microscope. After confirming the presence of a C-shaped canal, 3-D images were taken using CBCT as part of routine examination, diagnosis, and treatment planning. Bilateral molar data were also obtained to analyze the distribution of unilateral and bilateral occurrence of C-shaped canals, classified by Fan et al. (2004) [4]. Working lengths were measured using an apex locator (Root Zx mini, J Morita), cross-confirmed with pre-existing CBCT data. Canals were prepared using K files (Mani Ink Japan) and 4% and 6% NiTi rotary files if required. Throughout the preparation, all root canals were irrigated using 2 mL of 2.5% NaOCl solution after each instrument. After instrumentation, a final flush was administered using 5 mL of 17% ethylenediaminetetraacetic acid (EDTA, Smear Clear by SybronEndo, Orange, CA) for one minute and 5 mL of 2.5% NaOCl for one minute, followed by a final rinse with normal saline. Canals were obturated using E and Q plus thermoplasticized obturation. Two mesial and distal radiographs were taken, and canals were classified according to Melton et al. (1991) (see Figures 2-16) [7].

Statistical analysis

Statistical analysis examined the correlation between gender, the number of canals along root grooves, and C-shaped canals in mandibular second molars on one or both sides. A significance threshold of 5% (α = 0.05) was established. Given its suitability for categorical data analysis, the Chi-squared test was employed to analyze these connections. This method allows for examining relationships between variables and determining if observed differences are statistically significant. Additionally, insights into the correlation between the variables of interest were sought through this analysis.

## Results

Out of 200 mandibular second molars, 15 C-shaped canals were identified, with a roughly equal distribution across genders. The selected sample included 15 permanent mandibular second molars from eight males and seven females.

**Figure 17:**
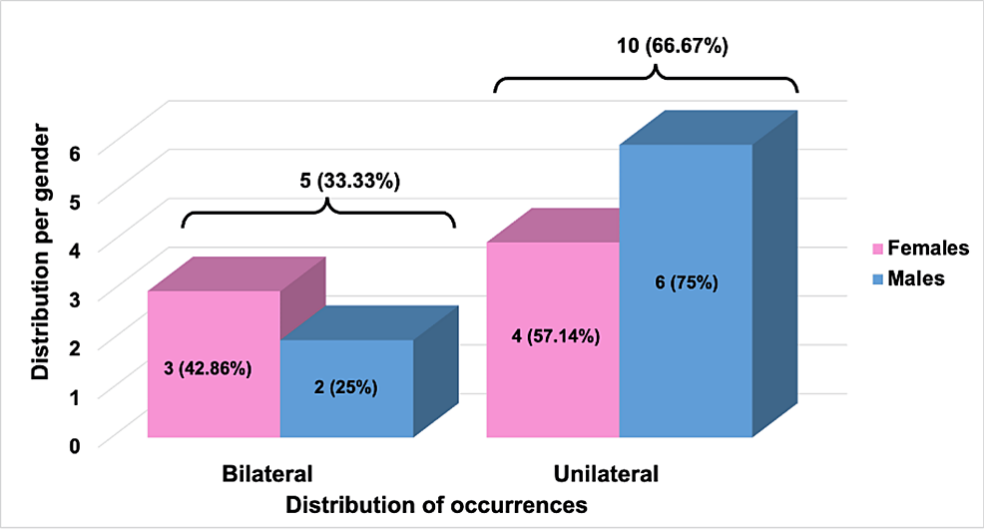
Distribution of occurrence (unilateral and bilateral) of C-shaped canals by gender The graph visually presents the prevalence of bilateral and unilateral occurrences among genders. Each bar, colored pink for females and blue for males, indicates the frequency of these occurrences. The actual occurrences count above each bar, representing absolute frequencies. Moreover, the percentages displayed on the bars reflect the relative proportion of each category compared to the total occurrences recorded. This dual representation enables understanding the absolute numbers and the proportional distribution of occurrences within each gender category.

Table 1 reports the prevalence of bilateral and unilateral conditions in a study population, broken down by gender. Among females (n=7), 42.86% experienced bilateral conditions, while the majority, 57.14%, had unilateral conditions. In contrast, a smaller proportion of males (n=8) had bilateral conditions at 25%, with a larger 75% experiencing unilateral conditions. Across the total sample (N=15), one-third (33.33%) had bilateral conditions, and two-thirds (66.67%) had unilateral conditions (see Figure 17). The Chi-square statistic of 0.535 with a p-value of 0.4645 suggests no significant difference in the distribution of the bilateral and unilateral conditions between genders. The percentages in parentheses indicate the subgroup's proportion within the gender category.

**Table 1:** Distribution of unilateral and bilateral C-shaped canals of patients by gender The table presents the frequency of bilateral and unilateral conditions among females and males. The sample sizes are seven females and eight males, with a total of 15 participants.

Gender	Bilateral	Unilateral	Chi-square Statistic	p-value
Females (n=7)	3 (42.86%)	4 (57.14%)	0.535	0.4645
Males (n=8)	2 (25%)	6 (75%)		
TOTAL (N=15)	5 (33.33%)	10 (66.67%)		

**Figure 18:**
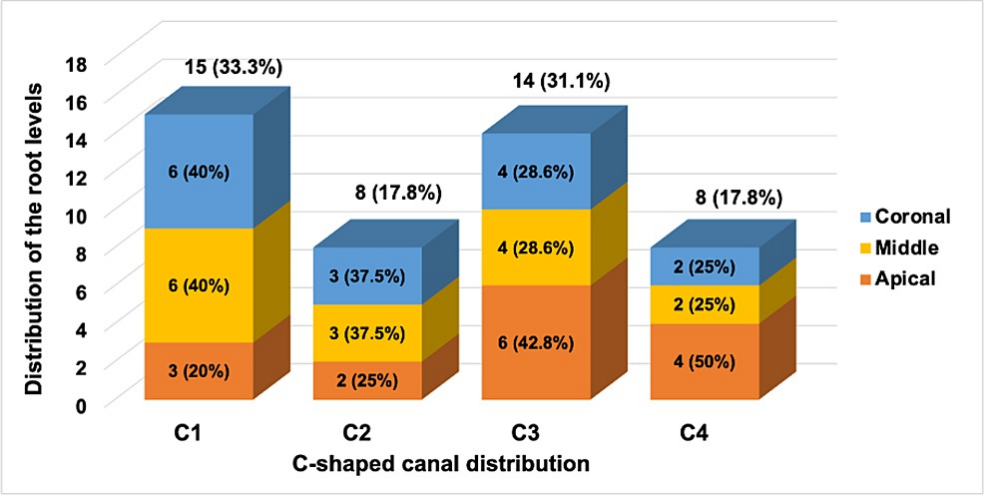
Distribution of the root levels in different C-shaped canal distribution The graph illustrates the distribution of C-shaped canals classified as C1 to C4 across different root levels within a tooth. These root levels are categorized as coronal (blue), middle (yellow), and apical (orange). For each canal configuration (C1 to C4), the graph displays the number and proportion of C-shaped canals at each root level.

Table 2 reports the distribution of the teeth with C-shaped canal systems with root levels. For canal configuration C1, occurrences were observed in 15 individuals (33.33%). Of these, six (40%) were at the coronal root level, six (40%) at the middle root level, and three (20%) at the apical root level. Canal configuration C2 was observed in eight individuals (17.8%), with three (37.5%) at the coronal root level, three (37.5%) at the middle root level, and two (25%) at the apical root level. Canal configuration C3 was exhibited by 14 individuals (31.1%), with four (28.6%) at the coronal root level, four (28.6%) at the middle root level, and six (42.8%) at the apical root level. Lastly, canal configuration C4 was demonstrated by eight individuals (17.8%), with two (25%) at the coronal root level, two (25%) at the middle root level, and four (50%) at the apical root level (see Figure 18). These findings provide insight into the distribution of anatomical features along different root levels, which can inform clinical approaches and treatment strategies tailored to specific root configurations.

**Table 2:** Distribution of the teeth with C-shaped canal systems with root levels The table presents the distribution of C-shaped canal systems at different root levels in mandibular second molars among the 15 individuals examined. Discrepancies were noted in the coronal, middle, and apical canal configurations.

Canal Configuration	Coronal	Middle	Apical	Chi-square Statistic	p-value
C1	6 (40%)	6 (40%)	3 (20%)	0.0821	3.023
C2	3 (20%)	3 (20%)	2 (13.33%)		
C3	4 (26.67%)	4 (26.67%)	6 (40%)		
C4	2 (13.33%)	2 (13.33%)	4 (26.67%)		

**Figure 19:**
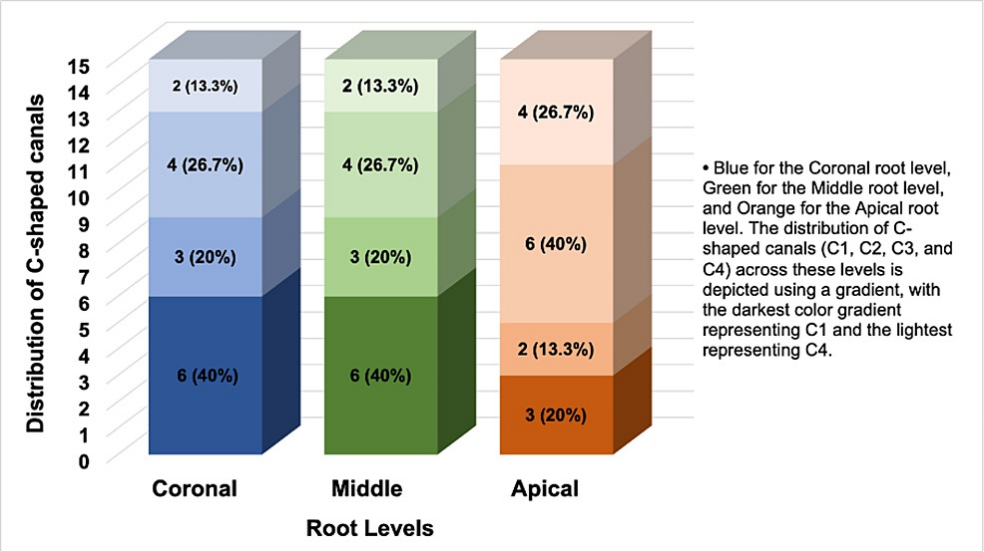
Distribution of the C-shaped canal distribution in root levels The graph provides a detailed visualization of C-shaped canals, categorizing them by their root level (coronal, middle, apical) and canal configuration (C1, C2, C3, C4). Each configuration is represented by a unique color gradient, progressing from dark to light to signify the classes from C1 to C4. The Y-axis indicates the canal count, while the X-axis depicts the root levels, color-coded with blue for the coronal, green for the middle, and orange for the apical levels. Additionally, numerical data and percentages are incorporated into the graph to thoroughly break down the distribution within each configuration and root level.

The distinct patterns of C-shaped canal distributions vary across various root levels of a tooth. At the coronal and middle root levels, the canal distributions were identical, with C1 canals being the most prevalent at 40%, followed by C3 canals at 26.7%, C2 canals at 20%, and C4 canals at 13.3%. However, a shift was observed at the apical root level, where the prevalence of C3 canals increased significantly to 40%, making them the most common at this level. C4 canals also increased to 26.6%, while C1 and C2 canals decreased to 20% and 13.3%, respectively (see Figure 19). This variation highlights a canal complexity and distribution change as the root canal progresses from the coronal to the apical end. This indicates a potential increase in treatment complexity and necessitates careful consideration in endodontic procedures.

Although canal configurations C1 and C2 were most frequently observed at the coronal and middle levels, whereas C3 was more prevalent at the apical level of the roots, the statistical analysis revealed no significant difference in the prevalence of C-shaped canals across root levels (p>0.05).

**Figure 20:**
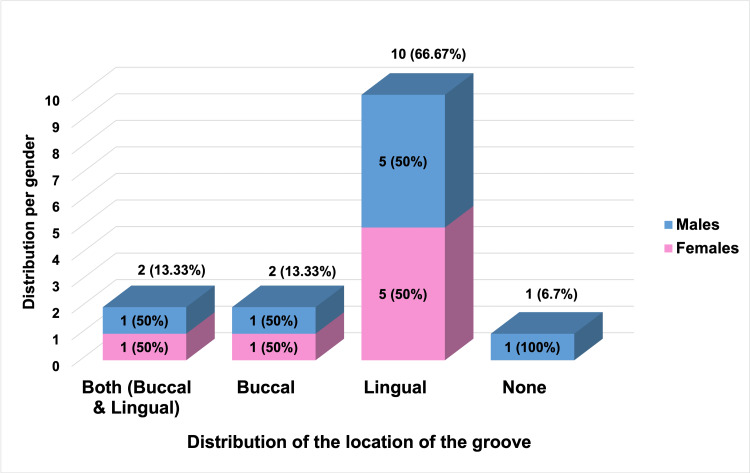
Distribution of the location of the groove in patients The graph illustrates a comprehensive analysis of groove distribution, distinguishing between buccal and lingual grooves and grooves exclusively on either the buccal or lingual side, as well as instances where no grooves are evident. Each bar in the graph is partitioned to depict genders - males in blue and females in pink. Moreover, the top of each bar showcases the overall percentages for each type of groove location, offering a quick overview of the prevalent groove patterns across genders.

Table 3 presents the distribution of the presence and location of grooves by gender among a sample of 15 individuals. Among females (n=7), grooves were most located lingually (71.4%), followed by an equal distribution between buccal and both buccal and lingual locations (14.3%) and buccal only (14.3%). No female participants were without a groove. In contrast, males (n=8) showed a more varied distribution, with lingual grooves being most frequent (62.5%), followed by both buccal and lingual (12.5%), and buccal only (12.5%). Additionally, 12.5% of males had no groove (see Figure 20). The incidence of grooves among patients exhibited a notable gender disparity, with a higher prevalence observed in males at 53% compared to females at 47%. Overall, the combined data (n=15) showed that lingual grooves were most prevalent (66.67%), followed by both locations (buccal and lingual) (13.33%), and then buccal only (13.33%). Only one individual (6.7%) lacked a groove. The Chi-square statistic of 0.938 with a p-value of 0.3328 indicates no significant difference in groove distribution between genders at the conventional 0.05 threshold. These findings have important implications for understanding C-shaped canal variations in mandibular second molars and can guide treatment planning and management in endodontic treatment.

**Table 3:** Distribution of presence of groove in patients with gender The table shows the percentage of female and male patients with buccal and lingual dental grooves. The Chi-square statistic and p-value indicate no significant gender difference in groove distribution. Percentages represent the proportion within each gender category.

Gender	Grooves				Chi-square Statistic	p-value
	Both (Buccal and Lingual)	Buccal	Lingual	None		
Females (n=7)	1 (14.3%)	1 (14.3%)	5 (71.4%)	0	0.938	0.3328
Males (n=8)	1 (12.5%)	1 (12.5%)	5 (62.5%)	1 (12.5%)		
TOTAL (N=15)	2 (13.33%)	2 (13.33%)	10 (66.67%)	1 (6.67%)		

## Discussion

Previously, most investigations on C-shaped canal anatomy relied on invasive studies using extracted teeth. Studies indicate that CBCT offers superior imaging capabilities to conventional medical computed tomography, with lower effective doses and shorter working times [8]. While many studies have utilized CBCT to illustrate root and canal anatomy across various populations, there is a paucity of comparative studies evaluating contralateral variability between mandibular second molars. The staining and clearing technique remains the primary method for evaluating the root canal system [9]. The surgical operating microscope significantly advances surgical endodontics. This innovation reflects a broader trend in medicine and dentistry towards precision and minimally invasive procedures, transforming the practice of endodontics and endodontic surgery. Operating microscopes play a crucial role in various aspects of treatment, from diagnosis and locating extra or missed canals to assessing the effectiveness of new cleaning and shaping systems and preventing or managing endodontic mishaps [10].

In this study, we evaluated 89 patients who sought endodontic treatment for mandibular second molars over 18 months at the Department of Conservative Dentistry and Endodontics, KIMSDU, Malkapur, India. Following access cavity preparations, the confirmation of C-shaped canals using a surgical endodontic microscope and CBCT imaging was performed for a comprehensive assessment of canal morphology and anatomy. Historically, Keith and Knowles (1911) were the first to depict a C-shaped root canal in the mandibular second molar [11].

Prevalence

Our discussion on the prevalence, classification, occurrence, and gender distribution of C-shaped canals reveals notable diversity across various global populations, underscoring the anatomical uniqueness inherent to specific geographic and ethnic backgrounds. Several studies (see Table 4) evidence this variability.

**Table 4:** Prevalence, classification, and occurrence of C-shaped canals in mandibular second molars in various regions The table displays the prevalence, classification, and occurrence of C-shaped canals in mandibular second molars in various regions, as reported by different studies. It includes data on unilateral or bilateral occurrences and the distribution across genders where available.

Authors	Region/Race	Prevalence of C-shaped Canal	Sample Size	Classification	Unilateral/Bilateral Occurrence	Gender
Mashyakhy et al. (2020) [12]	Saudi	7.90%	367 teeth; Yes, 29 (7.9%); No, 338 (92.1%)	C3 (35.6%); C1 (23.0%); C2 (20.7%); C4 (18.4%)	Right side absent (96.4%) present (3.6%); left side absent (94.8%) present (5.2%)	N/A
Ladeira et al. (2014) [13]	Brazil	15.30%	214 patients (406 teeth)	Three canals (43.5%)	Bilateral C-shaped (68.3%); Unilateral C-shaped (31.7%); two canals (37.1%)	Females (61.1%); Males (38.9%)
Yang et al. (2021) [14]	Korea	36.80%	1884 CBCT images of mandibular second molars	C1 (35.3%); C3b (21.6%); C2 (21.2%); C3a (17.5%); C4 (4.4%)	Bilateral C-shaped (85.9%)	Female (43.32%); Males (29.42%)
Zheng et al. (2011) [15]	China	38.60%	608 examined CBCT images	C1 (72.5%); C2 (18.1%); C3 (7.8%)	Bilateral C-shaped (81%); left side (12.5%); right side (6.3%)	N/A
Martins et al. (2016) [16]	Portugal	8.50%	1088 mandibular second molars	C3 (38.1%); C2 (23.4%); C1 (21.1%); C4 (17.2%)	Left side (7.6%); right side (9.5%)	Females (11.2%); Males (4%)
Kantilieraki et al. (2019) [17]	Greek	10.80%	524 mandibular second molars	C1 (77.4%); C3b (15.1%); C3a (7.5%)	Unilateral C-shaped (75.5%); bilateral C-shaped (24.5%)	N/A
Abdalrahman et al. (2022) [18]	Iraq	17.40%	368 mandibular second molars	C2 (56.3%); C3 (23.4%); C1 (15.6%); C4 (4.7%)	Unilateral C-shaped (35.9%)	N/A
Gomez et al. (2021) [19]	Venezuelan	19.50%	190 mandibular second molars	C3 (17.3%) in middle third; C1 (13.6%) in cervical third; C4 (12.7%) in the apical third; C2 (10%) in the middle third	N/A	Females (48.6%); Males (51.4%)
Kim et al. (2016) [20]	Korean	40%	1920 mandibular second molars	C1 (66%) at coronal; C2 (21%) at coronal; C3 (55.58%) at apical; C4 (12.08%) at apical	N/A	Females (54.14%); Males (39%)
Wadhwani et al. (2017) [21]	Indian	9.70%	238 CBCT scans	N/A	N/A	Females (11.3%); Males (7.3%)
Helvacioglu-Yigit and Sinanoglu (2013) [22]	Turkish	8.90%	271 mandibular second molars	C1 at the orifice level; C2 at the coronal level; C3 at the middle; C4 canal configuration at apical	N/A	N/A
Donyavi et al. (2021) [23]	Iran	9.20%	502 CBCT scans	N/A	N/A	N/A
Current Study	Indian (Western Maharashtra)	7.50%	200 mandibular second molars	C1 (33.33%); C2 (17.78%); C3 (31.11%); C4 (17.78%)	Unilateral C-shaped (60%); bilateral C-shaped (40%)	N/A

Mashyakhy et al. (2020) reported a 7.90% prevalence in Saudi Arabia, with C3 canals being most prevalent at 35.6% and a notably high rate of right-side absence at 96.4% [12]. Ladeira et al. (2014) in Brazil noted a 15.30% prevalence, with a majority exhibiting bilateral C-shaped canals (68.3%) and a higher occurrence in females (61.1%) [13]. In Korea, Yang SE et al. (2021) found a substantial 36.80% prevalence predominantly bilateral at 85.9% and more frequent in females (43.32%) [14]. The highest observed prevalence was in China at 38.60% (Zheng et al., 2011), showcasing a significant complexity with 72.5% being C1 canals [15]. Martins et al. (2016) in Portugal reported an 8.50% prevalence with a gender distribution leaning towards females (11.2%) over males (4%) [16]. From Greece, Kantilieraki et al. (2019) observed a 10.80% prevalence of mostly unilateral C-shaped canals [17]. In Iraq, Abdalrahman et al. (2022) reported a 17.40% prevalence with a notable 64.1% bilateral occurrence [18]. Venezuela's study by Gomez et al. (2021) showed a 19.50% prevalence with C3 canals predominantly in the middle third of the canal [19]. Korea again reported a high prevalence of 40% in Kim et al. (2016), with C1 canals at the coronal level affecting more females (54.14%) [20]. In India, Wadhwani et al. (2017) found a 9.70% prevalence with a higher incidence in females (11.3%), and Turkey reported an 8.90% prevalence (Helvacioglu-Yigit and Sinanoglu, 2013), analyzing the entire canal length from orifice to apical [21,22]. These findings illustrate anatomical variability and complexity and highlight gender-related differences in C-shaped canal prevalence. Our current study in Western Maharashtra, India, mirrors these findings with a 7.50% prevalence, offering crucial insights into the anatomical peculiarities necessitating tailored endodontic approaches specific to each demographic and regional population (see Table 4).

Moreover, according to the literature, the prevalence of C-shaped canals in mandibular second molars varies widely across different global studies, indicating significant anatomical diversity based on geographic and ethnic factors. For instance, Donyavi et al. (2019) reported a 9.20% prevalence in Iran, whereas Manning (1990) found a slightly higher prevalence of 12.80%, without specifying the regional demographic [23,24]. Rahimi et al. (2008) and Weine (1998) both observed a lower prevalence of 7.20% and 7.60%, respectively, indicating less frequent occurrences of this canal morphology in their studied populations [25,26]. Yang ZP et al. (1988) noted a prevalence of 13.90%, reflecting a moderate occurrence [27].

In contrast, a higher prevalence was noted by Haddad et al. (1999) at 19.10%, Gulabivala et al. (2001) at 22.40%, and particularly high by Seo (2004) at 32.70%, and Jin et al. (2006) who reported the highest at 44.50%, suggesting significant variations in specific populations, notably in Asian regions [28-31]. Al-Fouzan (2002) and another study by Gulabivala et al. (2002) reported a closer prevalence of 10.60% and 10%, respectively, reflecting a somewhat consistent observation in the Middle Eastern and Asian populations [32,33]. Lower prevalence rates were reported by Ahmed et al. (2007) and Peiris et al. (2007, 2008) in two studies with 10%, 6%, and 3%, respectively, highlighting less common occurrences in their respective study areas [34-36]. Al-Qudah et al. (2009) and Neelakantan et al. (2010) reported lower rates at 10.40% and 7.50% [9,37]. Zhang et al. (2011) and Wang et al. (2012) observed notably higher prevalence of 29% and 34.64%, respectively, in different Chinese populations, indicating a potential regional variation within China itself [38,39]. Lambrianidis et al. (2001) and Jung et al. (2010) reported some of the lowest and highest prevalences at 4.58% and 29%, suggesting that geographical and possibly genetic factors significantly influence the anatomical configuration of C-shaped canals [40,41]. These diverse findings emphasize the complex nature of C-shaped canal morphology and underscore the importance of considering regional and ethnic differences in dental practice, particularly in endodontics, where canal shape can significantly impact treatment outcomes.

Morphology of C-shaped roots and root canals

Various techniques have been employed to study the morphology of C-shaped roots and root canals. Traditionally, the clearing technique involves irrigation, drying, ink injection, sealing, dehydration, and observation under a stereomicroscope. Vertucci (1984) established a general system for root canal morphology using this method [42]. Other techniques include polyester resin cast replicas, histology, transmission electron microscopy, X-ray imaging, CT, and micro-computed tomography (micro-CT). While tooth morphology is typically bilaterally symmetrical, asymmetry cases have been reported. However, unusual root canal morphologies often occur symmetrically. Nakayama and Toda (1941) found a C-shaped root present bilaterally in 25% of reported cases [43].

Classification of C-shaped roots and root canals

Classification of C-shaped roots and root canals is crucial for understanding their variations and aiding in effective treatment. Typically, a C-shaped root canal is described as having a C-shaped configuration in cross-section, although it may not be continuous throughout. Various classification systems have been proposed to categorize C-shaped roots and root canals. Kotoku (1985) categorized C-shaped roots based on the depth of root grooves, while Carlsen (1990) classified them into three categories based on groove characteristics [44,45]. Melton et al. (1991) proposed a simplified classification system based on three categories: continuous C-shaped canal (category I), semicolon-shaped orifice with a mesial distinct canal (category II), and two or more separate canals (category III) with three subdivisions [7]. However, Fan et al. (2004) modified the classification system, which includes five categories (C1-C5) with detailed criteria [4]. Our study followed Fan et al.'s classification and observed canal configurations in 89 patients. The majority exhibited C1, C2, C3, or C4 configurations, with variations in distribution across the root canal system's coronal, middle, and apical thirds. Our analysis, facilitated by CBCT imaging, revealed shifts in canal configurations from the coronal to apical thirds, highlighting the dynamic nature of C-shaped root canal morphology [4,30,46].

Etiology of C-shaped roots and root canals

Takahashi et al. (1989) proposed that the thinner lingual dentine in C-shaped roots is due to slower dentine formation speed on that side, possibly caused by odontoblasts occupying a broader space [47]. They also suggested that reduced buccal cusp size in mandibular molars leads to furcation of lingual roots and non-furcation of buccal roots, contributing to C-shaped root canal formation. The gene(s) responsible for C-shaped roots in mice are located on chromosomes 5 or 17, although specific gene(s) have not been identified [48,49].

Identification of C-shaped root canals

Conventional intra-oral radiographs are commonly used to assess root canal anatomy, but panoramic radiography has shown good sensitivity and specificity in diagnosing mandibular molars with C-shaped root canals [41]. Despite its limitations, panoramic radiography is recommended as a routine step before endodontic treatment to determine the need for further examination.

Identifying C-shaped root canals on intraoral X-rays alone can be challenging, but clinicians should be familiar with their radiographic appearance. Haddad et al. (1999) noted the fusion or proximity of roots, a large distal canal, a narrow mesial canal, and a blurred image of a third canal in between on intraoral radiographs of semicolon-type C-shaped root canals [28]. Preoperative radiographs from multiple angles and additional imaging like bitewings, panoramics, or contra-lateral tooth radiographs aid identification. CBCT is increasingly used in endodontics due to its higher resolution and lower radiation dose than spiral CT. However, its use should be limited to cases where conventional radiography or alternative imaging methods are insufficient [50].

Endodontic treatment of C-shaped root canals

Effective root canal treatment of C-shaped canals starts with access cavity preparation, which is often crucial for success; using an operating microscope improves efficacy [51,52]. Guidelines from Krasner and Rankow (2004), based on 500 extracted human teeth, suggest principles like 'color change' and 'orifice location' for locating pulp chambers and root canal orifices, particularly useful for identifying C-shaped root canals in mandibular molars [53]. The cemento-enamel junction (CEJ) serves as a reliable landmark for access. Knowledge of pulp chamber anatomy helps prevent perforations. Modifications like a trapezoidal shape may be necessary for accessing additional canals, such as a second lingual canal [54,55]. To avoid perforations, the anti-curvature filing technique is recommended [56].

When cleaning C-shaped root canals, focus on the 'isthmus,' 'trough,' and 'fin' as potential bacterial reservoirs [3,57,58]. Ensure widening the isthmus without deepening towards the apex to prevent perforations [7,59]. Avoid strip perforation using files no larger than #25, and refrain from using Gates-Glidden burs on the mesiobuccal and buccal isthmus areas. Be cautious of root perforation, especially in thinner lingual walls of mandibular molars and mesial walls of mandibular first premolars [60]. Cleaning and irrigation with chemical solutions like sodium hypochlorite (0.5-5.25%) and EDTA are crucial. Ultrasonic equipment aids in cleaning fine root canals, but overuse can lead to perforation and file fracture. Light-activated disinfection and calcium hydroxide paste are also effective. Radiographs with small instruments inside canals are useful for confirming working length. Nickel-titanium (NiTi) rotary instruments reduce perforation risk but are insufficient to clean C-shaped root canals. Combining rotary and assisting instruments improves access to apical anatomy, enhancing treatment success.

Root canal filling is crucial for preventing reinfection by oral bacteria. Ordinola-Zapata et al. (2009) found similar gutta-percha filling percentages in Fan's radiographic types I-III at all root levels but lower in the apical third [61]. Various methods fill C-shaped root canals, including cold lateral condensation and warm vertical condensation with gutta-percha. The continuous wave condensation technique combines aspects of both. Our study applied different techniques based on canal configuration: cold lateral condensation for categories C1 and C4 and single cone sectional obturation for categories C2 and C3. Post-operative radiographs confirmed obturation, followed by composite permanent filling material for restorations.

Posts should ideally leave at least 1 mm of sound dentine around the canal [62]. Yet, in C-shaped root canals, the lingual wall averages 0.58 mm (± 0.21) and the buccal wall 0.96 mm (±0.26), making it challenging to maintain this thickness post-shaping [60,63]. Consequently, post-placement is generally not advised for C-shaped canals due to their dimensions [63]. However, the mesial root may be suitable, especially for circular posts. For post-preparation, it's recommended to focus on mesial or distal canals appearing circular [58,64].

In cases of failed root canal treatment in mandibular molars with C-shaped root canals, endodontic surgery presents challenges due to increased communication between root canals. Hemisection or root amputation is not recommended because these roots often lack a visible furcation ridge. Instead, extraction, root-end filling, and intentional replantation are advised when surgical intervention is necessary. Additionally, the conical shape of C-shaped roots generally facilitates easy extraction without fracturing.

The limitations of this study include a relatively small and geographically confined sample from Western Maharashtra, which may not be representative of other populations, limiting the generalizability of the findings. The cross-sectional study design precludes the establishment of causality or the assessment of changes over time. The reliance on specific imaging techniques, subject to interpretation biases, and the exclusion of certain complex cases due to stringent criteria may affect the comprehensiveness of the data. Additionally, the lack of longitudinal follow-up restricts insights into the long-term implications of C-shaped canals on treatment outcomes.

## Conclusions

This study demonstrated a 7.5% prevalence of C-shaped canals in mandibular second molars among the Western Maharashtra population, using endodontic microscopy and CBCT. No significant gender differences were observed in the prevalence of these anatomical features. CBCT and endodontic microscopy enhanced the visualization and understanding of this complex root canal morphology, facilitating more accurate diagnosis and treatment planning. The observed regional variation underscores the need for endodontic strategies tailored to specific anatomical complexities. The challenges associated with treating C-shaped canals highlight the importance of advanced imaging techniques in improving treatment outcomes. Future research should focus on the genetic and developmental factors influencing the formation of C-shaped canals. Longitudinal studies assessing the impact of different treatment modalities on clinical outcomes and investigations into the prevalence and characteristics of C-shaped canals in diverse populations are needed to enhance global endodontic practices.
